# Implantable Cardioverter-defibrillators for Primary Prevention in Pediatric Patients: What’s Next?

**DOI:** 10.19102/icrm.2018.090906

**Published:** 2018-09-15

**Authors:** George F. Van Hare

**Affiliations:** ^1^Department of Pediatrics, Washington University School of Medicine, St. Louis, MO, USA

**Keywords:** Children, ICD, long QT, sudden death

In this issue of *The Journal of Innovations in Cardiac Rhythm Management*, Dr. Townsend and Dr. Aziz have provided a very good review^1^ of the field of primary-prevention implantable cardioverter-defibrillators (ICDs) in children, focusing specifically on those with inherited arrhythmias. I am senior enough to have observed a progression of attitudes concerning primary prevention ICDs in children and, in particular, those with an established diagnosis of long-QT syndrome (LQTS). Initially, when ICDs were only available epicardially, via sternotomy or thoracotomy, we did not recommend them as primary prevention. Even with the arrival of the first transvenous systems, we were still quite hesitant, as we were uncertain as to whether they would be effective, given the prospect of possible electrical storm following an appropriate shock. Subsequently, when it became clear that ICDs were indeed effective in patients with LQTS,^2^ we did implant a number of ICDs for primary prevention. We now know, based on careful clinical studies in genotyped patients, that most patients with LQTS (particularly those with LQT1) do very well with appropriate β-blocker therapy.^3^ However, questions remain for the less common and less-well-studied diagnoses such as LQT3, Timothy syndrome, short-QT syndrome, and other rare mutations, for which the effectiveness of β-blockers has not been well-established.

As pediatricians, we are acutely aware that our patients have not yet survived childhood. This means that the experiences of our colleagues who see adult patients are not as informative for us as we would like them to be, as adult patients have self-selected, if you will, into a lower-risk category. Meanwhile, pediatric patients will (we hope) live for years or decades to come with the consequences of the decisions we make regarding device placement. It is well known that pediatric patients have a higher incidence of lead fracture^4^ as compared with adults, presumably due to their higher level of physical activity and the resultant stress on the lead components. Furthermore, the smaller the patient, the higher the likelihood of vein occlusion with the transvenous approach. Although lead extraction has come long way as a technique, with lower rates of morbidity, even the highest-volume centers continue to report mortality rates of 0.5% to 1% for the procedure.^5^ As pediatricians, we need to be circumspect and recognize that each time we place a transvenous lead, we are most likely setting our patients up for the need for eventual lead extraction.

So, where does this all leave us? I would argue that these considerations should prompt us to try to push up the lower age limit for transvenous system implantation, so as to lower the likelihood that our pediatric patients will require lead revision due to growth or fracture. We should also be more aggressive with respect to the development of epicardial hybrid systems, which can now be placed with small incisions and which seem to work well.^6^ Finally, we should lobby for improved subcutaneous ICD systems with fewer associated cosmetic issues and much lower rates of inappropriate shocks.
